# Telomeres and viruses: common themes of genome maintenance

**DOI:** 10.3389/fonc.2012.00201

**Published:** 2012-12-31

**Authors:** Zhong Deng, Zhuo Wang, Paul M. Lieberman

**Affiliations:** The Wistar InstitutePhiladelphia, PA, USA

**Keywords:** virus, telomere, replication, EBV, KSHV, HHV6, MDV

## Abstract

Genome maintenance mechanisms actively suppress genetic instability associated with cancer and aging. Some viruses provoke genetic instability by subverting the host’s control of genome maintenance. Viruses have their own specialized strategies for genome maintenance, which can mimic and modify host cell processes. Here, we review some of the common features of genome maintenance utilized by viruses and host chromosomes, with a particular focus on terminal repeat (TR) elements. The TRs of cellular chromosomes, better known as telomeres, have well-established roles in cellular chromosome stability. Cellular telomeres are themselves maintained by viral-like mechanisms, including self-propagation by reverse transcription, recombination, and retrotransposition. Viral TR elements, like cellular telomeres, are essential for viral genome stability and propagation. We review the structure and function of viral repeat elements and discuss how they may share telomere-like structures and genome protection functions. We consider how viral infections modulate telomere regulatory factors for viral repurposing and can alter normal host telomere structure and chromosome stability. Understanding the common strategies of viral and cellular genome maintenance may provide new insights into viral–host interactions and the mechanisms driving genetic instability in cancer.

## INTRODUCTION

Repetitive DNA elements provide essential functions in genome maintenance. The repetitive DNA elements at the ends of linear genomes have been recognized for their special role in preventing DNA loss due to the “end-replication problem” (Watson, 1972; Olovnikov, 1973). In most eukaryotes, the DNA repeats at the ends of linear chromosomes are referred to as telomeres and have essential functions in chromosome end-protection and genome stability (reviewed in Cech, 2004; Blackburn et al., 2006). Similar to cellular genomes, many DNA viruses have terminal repeats (TRs) that are essential for viral genome stability. Indeed, viral-like elements have been proposed to be the evolutionary source of cellular telomeres and telomerase (Nosek et al., 2006). For both viruses and cellular genomes, the function and regulation of these repetitive elements play a critical role in genome maintenance.

Most eukaryotic chromosomes have short (5–10 nucleotide) GC-rich telomere repeat elements that are essential for maintaining the linear structure of the chromosome. Telomere repeats can form structured DNA, like G-quadruplexes, that may provide structural stability to prevent nucleolytic degradation (Huppert, 2008; Qin and Hurley, 2008). Telomere repeats can also serve as binding sites for proteins that physically cap the ends of linear chromosomes and facilitate end-replication (de Lange, 2005a; Palm and de Lange, 2008). A minimal number of telomere repeats is required for end-protection, and repeat copy number can be amplified by specialized mechanisms that include telomerase-dependent reverse transcription (Cech, 2004; Chan and Blackburn, 2004), homologous recombination (McEachern and Haber, 2006; Cesare and Reddel, 2008), and in some organisms, telomere-specific retrotransposition (Silva-Sousa et al., 2012; Zhang and Rong, 2012). Telomere repeats can also function in transcription regulation (Arnoult et al., 2012), chromatin packaging (Schoeftner and Blasco, 2009; Ye et al., 2010b), subcellular localization (Mai and Garini, 2006), and chromosome segregation (Houghtaling et al., 2011).

Repetitive DNA elements play a significant role in viral genome biology and maintenance. For linear DNA viruses, TRs are required for viral genome stability. Functions of viral TRs include replication initiation, transcription regulation, integration, transposition, segregation, and virion packaging. Like telomeres, viral TRs can vary in size, composition, and copy number. Viral TRs bind to host and viral proteins, and these protein–DNA interactions are important for viral replication and genome maintenance. The mechanisms that regulate viral TR homeostasis may be similar to that of cellular telomere repeat copy number maintenance, but viral-specific nuances and limited experimental data may limit the extent of the comparison with cellular processes.

Viral infection can have profound effects on host cell processes, including those relevant to telomere biology and genome maintenance. Viruses that induce host cell proliferation and immortalization typically induce telomerase and prevent telomere shortening to escape senescence (Bellon and Nicot, 2008). Linear DNA viruses encode factors that alter DNA damage recognition and end-repair that can alter host telomere maintenance. Even circular viruses can utilize telomere repeat factors for viral genome maintenance, and indirectly modulate host telomere functions. Here we review some of the common features of viral and cellular genome maintenance elements, and how virus infections can alter host cell telomere maintenance.

## TERMINAL STRUCTURE OF VIRAL GENOMES

**FIGURE 1:**
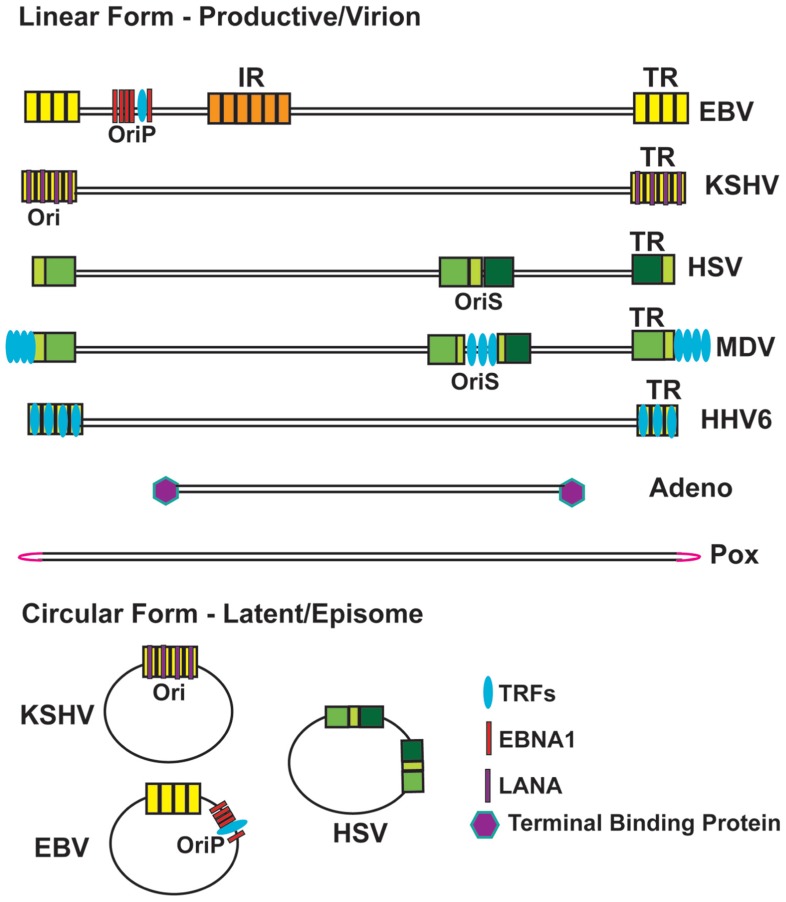
**Schematic of Viral Genome Terminal Repeat Structure in Linear and Circular Conformations**. Viral terminal repeats (TR) and intergenic repeats (IR) are shown as various colored box, indicating different repetitive sequences. Adenovirus TP and LANA bind to TR of adenovirus and KSHV, respectively. EBNA1 binds to both FR and DS region of EBV OriP. TRFs bind to DS region of EBV OriP and MDV OriS, and TRFs-binding sites are indicated for TR of MDV and HHV6. The terminal hairpin structure for Pox virus is indicated in pink.

All linear DNA viruses have specialized mechanisms for genome end-protection (**Figure 1**). Pox viruses are large (~250 kb) double-stranded DNA molecules with TRs that are covalently closed hairpins (Traktman and Boyle, 2004). Some prokaryotic pathogens, including the spirochete *Borrelia* that causes Lyme disease, have a similar terminal hairpin structure (Chaconas and Kobryn, 2010). Both genomes encode a topoisomerase-like resolvase (A22 for vaccinia and Res T for *Borrelia*) that cleaves the terminal hairpin during DNA replication. Pox viruses are also unusual in that they replicate their DNA genomes in the host cytoplasm. The cytoplasmic viral genomes may gain additional protection by forming specialized replication compartments consisting of viral-encoded proteins (Novoa et al., 2005). Similar protective replication compartments are observed in the nucleus for some viral genomes (e.g., herpesviruses) and may also occur at cellular sites of replication and repair.

Adenoviruses enter the nucleus as linear genomes with inverted TRs of ~100 bp that covalently bind to the viral terminal-binding protein (TP) during viral replication (de Jong et al., 2003). Adenovirus TP forms a covalent tyrosine hydroxyl linkage to DNA, mechanistically related to the action of topoisomerases and tyrosine recombinases (Yang, 2010). Covalently bound TPs have been described in prokaryotic linear genomes of *streptomyces* and prophage N15 (Huang et al., 2007). Terminal-binding proteins provide torsional strain and membrane anchoring in some organisms (Tsai et al., 2010). Topoisomerases, which modulate torsional strain, have specialized functions in host cell telomere DNA replication and DNA end-protection (Temime-Smaali et al., 2008; Germe et al., 2009; Ye et al., 2010a). Whether cellular topoisomerases function as end-binding proteins at cellular telomeres during DNA replication remains an intriguing possibility.

Herpesviruses enter cells as linear genomes with GC-rich TRs of variable length. Herpesvirus TRs are essential for multiple aspects of the viral life cycle, including gene expression, DNA replication, and recombination. The TRs of all herpesvirus genomes contain recognition sites for terminase, a viral-encoded endonuclease that generates a unit length linear form of the genome prior to packaging in the viral capsid (Zimmermann and Hammerschmidt, 1995; Bogner, 2002; Nadal et al., 2010). Interestingly, herpesvirus terminases have RNaseH/integrase-like folds and can be inhibited by anti-HIV drugs that target integrase (Nadal et al., 2010). The TRs can expand or contract upon lytic replication, and the copy number variation can be used as a measure of replication and clonality (Raab-Traub and Flynn, 1986). The mechanism regulating TR expansion, copy number control, and fusion are not fully understood.

Some herpesvirus members [e.g., Epstein–Barr virus (EBV) and Kaposi’s sarcoma-associated Herpesvirus (KSHV)] circularize upon entry in the nucleus, and form stable minichromosomes capable of long-term maintenance (**Figure 1**). The circular genomes fuse at the TRs and the circular genomes retain variable numbers of these repeats. Genome circularization is one mechanism through which linear chromosomes can protect their ends from exonucleolytic attack. In yeast, telomere repeat loss is rescued by chromosome circularization (Natarajan and McEachern, 2002; Tomaska et al., 2004). Stable circular human chromosomes can also be observed in rare ring-syndromes, but the genetic basis for this remains unknown (Le Caignec et al., 2004). In mammalian cells, chromosomes with critically few telomere repeats form inter-telomere fusions (de Lange, 2002; De Lange, 2005b; Bailey and Murnane, 2006; Bhattacharyya and Lustig, 2006). Telomere fusions in mammalian cells can occur through RAD52-dependent homologous recombination, or more commonly, through Ku-dependent non-homologous end-joining (Murnane, 2011). The mechanism of herpesvirus circularization depends on non-homologous end-joining enzymes DNA Ligase IV and XRCC4 (Muylaert and Elias, 2007), as well as chromosome condensation protein regulator of chromosome condensation 1 (RCC1; Strang and Stow, 2007), but the molecular details of viral circularization remains to be determined.

Selective integration into host telomeric DNA appears to be a common target site for some herpesvirus family members. Human Herpesvirus 6 (HHV6) and Marek’s disease virus (MDV) have TTAGGG repeats identical to host cell telomere repeats at the ends of their linear genomes (Arbuckle and Medveczky, 2011). These telomere repeats facilitate integration and mobility into host cellular telomeres during viral latency (Arbuckle et al., 2010; Kaufer et al., 2011b). In addition, MDV encodes a telomerase-like RNA that can interact with host cell telomerase, but it is not clear how this modulates telomerase activity, or whether it promotes viral integration at telomeres (Kaufer et al., 2010, 2011a). HHV6 may encode a replicase similar to adeno-associated virus (AAV), a parvovirus that integrates into a specific sequence in chromosome 19. Targeted integration into the telomere repeats appears to be mediated by homologous recombination with genome ends, but telomere targeting may be mediated by other mechanisms, like those that direct transposition in *Drosophila* telomeres.

## TELOMERIC FACTORS THAT RECOGNIZE AND REGULATE VIRAL GENOME MAINTENANCE

Telomere repeat-binding factors (TRFs), including all components of Shelterin, play a critical role in coordinating telomere repeat number with telomere end-protection, DNA replication, and DNA damage response (de Lange, 2005a; Palm and de Lange, 2008). Telomere repeat factors interact with numerous components of the DNA damage signaling pathways, as well as with components of DNA replication and chromatin assembly. As mentioned above, several viral genomes contain telomere repeat sites, most notably HHV6 and MDV, which contain TTAGGG-TRs. The TRs of these viral genomes do not appear to provide episomal stability (Bulboaca et al., 1998), but can direct viral genomes toward host telomere integration during latency (Arbuckle and Medveczky, 2011; Arbuckle et al., 2010; Kaufer et al., 2011b). TRF1 and TRF2 have been suggested to play a role in the integration process through binding to the TRs. While TRF2 prevents cellular telomere end-to-end fusions (Denchi and de Lange, 2007), it is possible that viral infection alters TRF function to promote viral integration by homologous recombination. Other viruses, like EBV, have functional monomeric TRF-binding sites within the episome maintenance element, OriP (Deng et al., 2002, 2003). OriP is an internal repeat element that consists of a family of repeats (FRs) and a dyad symmetry (DS) element, both of which bind to the viral-encoded episome maintenance protein EBV nuclear antigen 1 (EBNA1). The DS element is remarkable for its capacity to initiate bidirectional DNA replication in an EBNA1- and origin recognition complex (ORC)-dependent manner. The DS recruits ORC, and TRF2 facilitates and enhances this recruitment (Deng et al., 2002, 2003; Atanasiu et al., 2006). Disruption of TRF-binding in DS compromises ORC recruitment, DNA replication, and episome maintenance of OriP.

Studies from our lab indicated that TRF2 amino terminal basic domain contributes to ORC recruitment at EBV OriP (Deng et al., 2002, 2003). TRF2 was also found to recruit ORC to a subset of cellular telomeres. The TRF2 basic domain was found to be similar to the EBNA1 linking region, which contain RGG-like motifs that have been implicated in both metaphase chromosome attachment (Nayyar et al., 2009; Sears et al., 2003, 2004) and RNA-binding (Snudden et al., 1994). Investigation of the RNA-binding activity revealed selective interaction with single-stranded RNA oligonucleotides capable of forming G-quadruplex structures (Biffi et al., 2012; Norseen et al., 2009). Neither the EBNA1 nor TRF2 RGG-motifs bound single-stranded DNA oligonucleotides with G-quadruplex forming capacity. RNA-binding was also shown to facilitate interaction with ORC for both EBNA1 and TRF2. RNase A treatment reduced EBNA1 recruitment of ORC at OriP and EBNA1 association with mitotic chromosomes suggesting that RNA-binding was important for viral genome replication and episome maintenance.

While the endogenous RNAs bound by EBNA1 and TRF2 have not been fully characterized, both EBNA1 and TRF2 bound to telomere repeats-containing RNA (TERRA) with high affinity using *in vitro* binding assays including RNA pull-down assays and EMSA (Deng et al., 2009). Endogenous TERRA bound most efficiently to TRF2 and TRF1 using RNA-ChIP assays. In contrast, EBNA1 did not bind efficiently to endogenous TERRA, but does interact efficiently with viral-encoded EBV-encoded RNA (EBER) small non-coding RNAs expressed in close proximity to OriP (Snudden et al., 1994; Lu et al., 2004). The role of RNA-binding by EBNA1 and TRF2 in ORC recruitment is not completely clear. Depletion of TERRA RNA using siRNA resulted in a change in histone modifications within the telomere repeats and adjacent subtelomeric regions. TRF2 and TRF1, as well as their counterparts in different species, have been implicated in telomere replication, and it remains possible that RNA-binding and interactions with ORC play a significant role in telomere chromatin structure and regulation.

## CHROMATIN STRUCTURE OF VIRAL TERMINI

**FIGURE 2:**
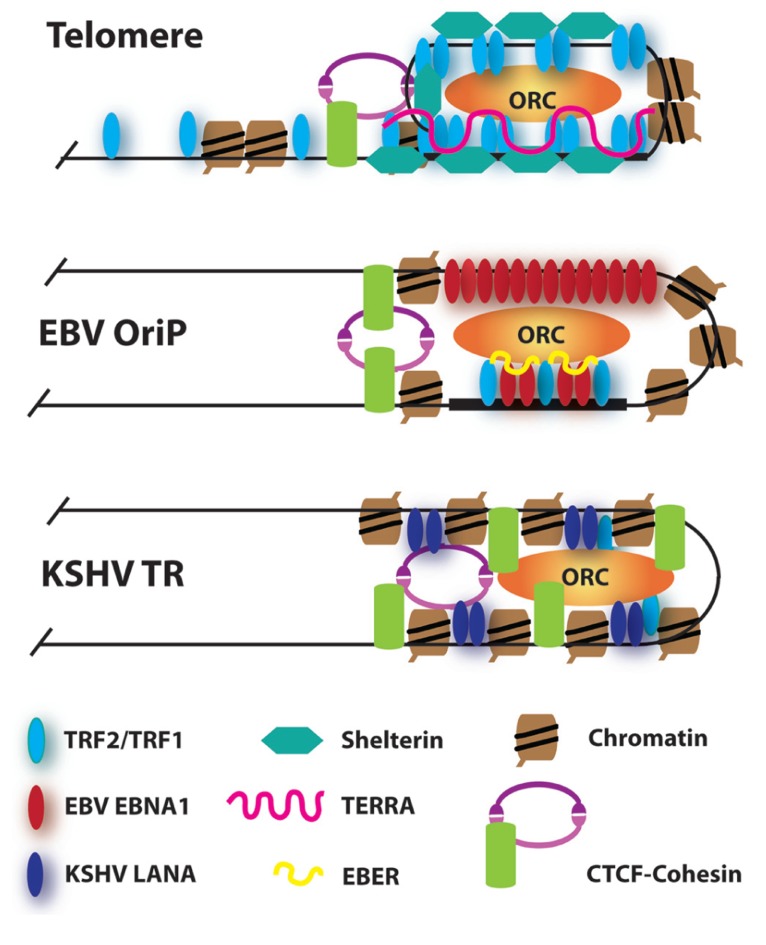
**Model of higher order chromatin structures at telomeres and viral maintenance elements**. RNA-dependent recruitment of ORC at telomeres and EBV OriP is indicated. Elevated histone H3K4me3 and CTCF-cohesin enrichment is found at all three maintenance elements**. **TRFs are localized to the latent origin of both EBV and KSHV.

The chromatin structure of viral maintenance elements may share common features with telomeric chromatin (**Figure 2**). Telomeric chromatin is highly dynamic and can adopt multiple conformations to coordinate cell cycle regulated changes in transcription and DNA replication (Cesare and Karlseder, 2012). Transcription of TERRA may facilitate telomere DNA replication, as well as promote subsequent heterochromatin formation. TRF2 and TRF1 binding to TERRA can stabilize ORC-binding and ORC-associated heterochromatin at telomeres (Deng et al., 2009). At EBV OriP, EBNA1 and TRF2 may bind to viral-encoded EBER RNA, rather than TERRA, to recruit ORC (Norseen et al., 2008). ORC is recruited to the KSHV TR through interactions with latency-associated nuclear antigen (LANA), but it is not known if this interaction has an RNA-binding component (Stedman et al., 2004). LANA binds to KSHV TRs, and functions, like EBV EBNA1, to tether the viral genome to metaphase chromosomes. In contrast to EBNA1, LANA targets metaphase chromosomes through interactions with histone H2A/H2B (Barbera et al., 2006). LANA also interacts with other host chromatin factors, including ORC, BRD2/4, DEK, p53, and DNMT3a, which may affect chromatin structure and maintenance of the TR (Ballestas and Kaye, 2011; Verma et al., 2007). These comparative studies suggest that ORC and heterochromatin formation play a central role in genome maintenance function.

Recent studies have also implicated CCCTC-binding factor (CTCF) and cohesin in the higher order chromatin structure of telomeres and viral maintenance elements (Deng et al., 2012). CTCF and cohesin were found to bind to the majority of human subtelomeres in close proximity to the presumptive start sites of TERRA transcripts. CTCF and cohesin have been shown to bind to regions surrounding EBV OriP and mediate long-distance enhancer–promoter regulatory interactions and chromatin boundary functions (Tempera et al., 2010, 2011). Nucleosome mapping studies indicate that histones are strongly positioned at sites adjacent to the EBV and KSHV maintenance elements (Zhou et al., 2005). The positioned histones are elevated in H3K4me3, which is also elevated among histones neighboring the CTCF sites in human subtelomeres. Higher order chromatin structure may also form at the EBV TRs, and mediated, in part, through binding sites for Pax5 (Arvey et al., 2012), a cellular factors implicated in chromatin condensation during immunoglobulin gene rearrangements in B-lymphocytes (Fuxa et al., 2004). These observations suggest that viral maintenance elements and telomeres may adopt similar higher order chromatin structures, which may facilitate mobilization and re-localization to subcellular domains.

Viruses and telomeres can colocalize at common subnuclear structures, including nuclear pores, nuclear periphery, and promyelocytic leukemia (PML) nuclear bodies (PML-NBs). PML-NBs have been implicated in anti-viral functions, as well as in chromatin repression, and telomere recombination (Henson et al., 2002; Everett and Chelbi-Alix, 2007; Brouwer et al., 2009; Draskovic et al., 2009; Chung et al., 2012). The primary cellular constituents of PML-NBs, including PML, SP100, death-domain associated protein (Daxx), and alpha thalassemia/mental retardation syndrome X-linked (ATRX), function in chromatin assembly and regulation. Daxx is commonly associated with histone deacetylases (HDACs) and ATRX is a histone H3.3 chaperone with SNF-like ATPase remodeling activity. Recent studies have implicated ATRX in the deposition of H3.3 at telomere repeats and other GC-rich repetitive DNA elements (Goldberg et al., 2010; Lewis et al., 2010). Cells lacking or depleted in ATRX have an increase in TERRA abundance, indicating that ATRX is involved in transcriptional repression at telomere repeat DNA (Goldberg et al., 2010). ATRX and Daxx are known to repress viral transcription and replication, but other than recruitment of HDACs, little is known about the mechanism of viral genome repression. Sequestration of GC-rich repetitive regions may be a common function for PML-NBs, but it is also possible that free DNA ends require specialized histone chaperone and assembly machinery. Most DNA viruses encode proteins that disrupt or alter the function of PML-NBs and their various components (Tavalai and Stamminger, 2009). Herpes simplex virus (HSV) encodes ICP0, which functions as an E3 ubiquitin ligase that targets PML degradation (Everett and Chelbi-Alix, 2007). Human cytomegalovirus (hCMV) encodes tegument protein pp71 that degrades Daxx (Hwang and Kalejta, 2009), and EBV encodes EBV major tegument protein (BNRF1) protein that disrupts ATRX interaction with Daxx (Tsai et al., 2011). These viral proteins may be predicted to affect telomere chromatin and transcription regulation, but it is not clear if they selectively target chromatin at viral termini rather than cellular telomeres.

## TRANSCRIPTION OF VIRAL TERMINI

Transcription of viral and cellular TRs may contribute to genome maintenance and stability. Transcription of cellular telomeres has been detected in almost all organisms where it has been investigated. TERRA is expressed from multiple different telomeres in a largely heterogeneous manner (Azzalin et al., 2007; Schoeftner and Blasco, 2008). The regulation and function of telomeric RNA has been reviewed in detail elsewhere (Arora et al., 2011; Chawla and Azzalin, 2008; Feuerhahn et al., 2010). The TRs of several viruses can be transcribed, potentially generating transcripts similar to TERRA. The terminal TTAGGG repeats of HHV6 and MDV have the potential to generate viral TERRA, but this has not yet been experimentally identified. It is also possible that viral genomes integrated in cellular telomeres can regulate telomere transcription and chromatin. Reactivation of latent virus that is integrated into viral telomeres may correlate with activation of viral TTAGGG transcription.

The TRs of EBV can be transcribed, but only after genome circularization. Genome circularization generates the template required for the viral-encoded proteins LMP2a and LMP2b (Laux et al., 1989). Latent membrane protein 2 (LMP2) promoter is located in the unique right region of the viral genome, and transcription proceeds rightward across the TR junction and continues into the fused unique left region of the viral genome. LMP2 is a highly spliced mRNA, and the TRs themselves do not contribute to the open reading frame. LMP2 provides an important B-cell survival function, as well as inhibits viral lytic cycle reactivation (Brinkmann and Schulz, 2006; Longnecker, 2000; Rechsteiner et al., 2008). It may be possible that genome circularization and LMP2 template formation is coordinately regulated with host-cell growth and survival pathways.

The TR of HSV encode latency-associated transcript (LAT), the primary transcript expressed during latent infection in neuronal ganglia (Bloom, 2004). The full length LAT is generated from a fused or circularized junction of viral TRs, similar to the TR template for EBV-encoded LMP2. The LAT transcript is processed into a stable 2.0 kb intron and several miRNAs (Atanasiu and Fraser, 2007; Umbach et al., 2008). The LAT transcript provides an anti-apoptotic activity to the latently infected neuronal cells (Perng et al., 2000), and at least one miRNA that suppresses viral lytic cycle reactivation (Umbach et al., 2008). The LAT transcript may also interact with chromatin regulatory factors, including members of the polycomb family, which may regulate viral genome stability during latent infection (Kwiatkowski et al., 2009).

## DNA REPLICATION OF REPETITIVE ELEMENTS

Telomere DNA replication has been reviewed comprehensively elsewhere (Chakhparonian and Wellinger, 2003; Gilson and Geli, 2007; Verdun and Karlseder, 2007; Cesare and Reddel, 2008; Ye et al., 2010b; Stewart et al., 2012). We consider here only a few aspects of telomere replication that reflect the relationship between virus and host genome maintenance. As mentioned above, both EBV and KSHV maintenance elements efficiently recruit ORC. Nevertheless, replication can initiate at sites outside of these origins (Norio and Schildkraut, 2004; Verma et al., 2011). ORC-binding sites have been mapped to the subtelomeric X and Y’ elements of *Saccharomyces cerevisiae*, but replication initiation may not occur frequently at these potential origins. ORC can also bind to host chromosome regions enriched in telomeric repeat DNA (Deng et al., 2007). However, initiation of DNA replication occurs infrequently at telomere repeats (Sfeir et al., 2009), and appears to initiate primarily within the large subtelomeric regions (Drosopoulos et al., 2012). These findings suggest that origin function at these sites is auxillary, and that the primary function of ORC recruitment is in heterochromatin formation and DNA repeat stability (Prasanth et al., 2010; Chakraborty et al., 2011).

Telomere repeat-binding factors may play a role in coordinating replication with recombination. Myb-family proteins, like TRF1 and TRF2, may have intrinsic capacity to modulate DNA polymerase progression. *In vitro*, both TRF1 and TRF2 stall DNA replication forks (Ohki and Ishikawa, 2004). However, *in vivo* TRF1 prevents replication fork stalling and facilitates telomere DNA replication (Sfeir et al., 2009); TRF2 also contributes to efficient telomere replication *in vivo* by regulating topological stress (Ye et al., 2010a). Similarly, the fission yeast telomere repeat factor Taz1 promotes DNA replication through telomere repeats, potentially suppressing DNA secondary structures that block polymerase processivity (Miller et al., 2006; Dehè et al.,2012). In contrast, some myb family members, like REB1 and RTF1 in budding yeast, functions as a replication fork blocking protein that regulate DNA catenation and replication termination (Biswas and Bastia, 2008; Eydmann et al., 2008). Replication fork regulation may play important roles in controlling recombination and sister-chromatid cohesion, both of which are critical for viral and cellular genome maintenance.

The viral encoded origin-binding proteins for EBV and KSHV, EBNA1 and LANA, also cause replication fork stalling (Dheekollu and Lieberman, 2011). Recent studies indicate that replication fork stalling result in the recruitment of the replisome protection factor Timeless (Dheekollu et al., 2011). Timeless is the human ortholog of the *Saccharomyces cerevisiae* Tof1 and the *Schizosaccharomyces pombe* Swi1. Its function in replication fork protection appears to be conserved. Recently, TRF1 has been shown to interact with Timeless at mammalian telomeres and was required for telomere length maintenance and integrity (Leman et al., 2012). Replisome protection may be required to prevent loss of repeat elements during semi-conservative replication. Timeless has also been shown to contribute to sister-chromatid cohesion in mammalian cells (Leman et al., 2010; Dheekollu et al., 2011). Sister-chromatid cohesion may be important for repeat stability, but may also contribute to faithful chromosome segregation. Thus, viral episome maintenance elements may utilize telomeric mechanisms for DNA replication and sister-chromatid cohesion.

## RETROTRANSPOSITION: A VIRAL-MECHANISM OF TELOMERE MAINTENANCE

Retrotransposons are endogenous retroviral-like DNA elements that drive genome diversification during evolution (Burns and Boeke, 2012; Silva-Sousa et al., 2012). In *Drosophila*, telomeres consist of retrotransposons that modulate chromosome length by site-specific transposition at the termini (Zhang and Rong, 2012). In *Bombyx mori*, transposition occurs within a pentameric telomere repeat and may compete with telomerase elongation mechanisms (Tatsuke et al., 2009; Osanai-Futahashi and Fujiwara, 2011). Site-directed retrotransposition is thought to involve an RNA-binding and nuclear import activity of the GAG protein that is then directed to the site of RNA origination. Although there is no evidence for retrotransposition as a mechanism of mammalian telomere maintenance, it is interesting to note that TERRA transcripts are retained at telomeres through interactions with telomere-associated proteins, including TRF2 and TRF1 (Deng et al., 2009; Biffi et al., 2012). Retention of TERRA RNA at telomeres is likely to influence DNA replication, either through the direct inhibition of telomerase (Schoeftner and Blasco, 2008; Redon et al., 2010), or by controlling resection by nucleases like ExoI (Pfeiffer and Lingner, 2012). In ALT cells, TERRA may contribute to recombination-based telomere elongation, but details of this potential mechanism have not been characterized completely. As mentioned above, virus replication mechanisms may utilize RNA-facilitated DNA recombination (Rennekamp and Lieberman, 2010, 2011). Thus, components of RNA-directed replication that occurs in lower eukaryotic retrotransposons, may be retained in telomere maintenance mechanisms in higher eukaryotes and their viruses.

## VIRAL MODULATION OF HOST TELOMERE MAINTENANCE

Immortalizing viral infections rewire host control of the cell cycle and DNA replication, including telomerase activation and telomere elongation. Most of the known viral mechanisms for telomerase activation involve transcriptional activation of human telomerase reverse transcriptase (hTERT). Human papillomavirus (HPV) E6 activates hTERT transcription through a cMyc-dependent pathway (Moody and Laimins, 2010), while KSHV LANA activates hTERT through Sp1 (Verma et al., 2004). EBV activates hTERT in two stages, the first following B-cell proliferation, and the second as a bypass to crisis-associated cellular senescence (Sugimoto et al., 2004; Takahashi et al., 2003). At least one EBV encoded protein, latent membrane protein 1 (LMP1), activates hTERT through the NF-κB pathway (Terrin et al., 2008). For EBV, lytic reactivation typically occurs in response to various cellular stresses, and typically requires cell cycle arrest. It is therefore interesting that hTERT was found to inhibit EBV lytic replication (Terrin et al., 2007). This suggests that telomerase activation status may modulate viral infection and replication, just as viral infection affects telomerase activation.

Non-immortalizing viruses may also affect telomerase activity of the infected cell. hCMV has been reported to induce telomerase through induction of hTERT transcription during primary infection of human diploid fibroblasts (Straat et al., 2009). This has been proposed as a potential mechanism of hCMV carcinogenesis. Chromosome instability and telomere shortening have been reported in cells chronically infected with hepatitis B virus (HBV) and in HBV-associated hepatocellular carcinoma (HCC; Lee et al., 2009). This correlated with an upregulation in shelterin proteins TRF1 and TRF2 in HCC foci (Oh et al., 2005). The chronic infection associated with HCV, an RNA virus, has also been reported to affect telomerase. The HCV core particle can increase telomerase nuclear localization and activity when co-expressed in hepatocellular carcinoma cell lines (Zhu et al., 2010).

**FIGURE 3:**
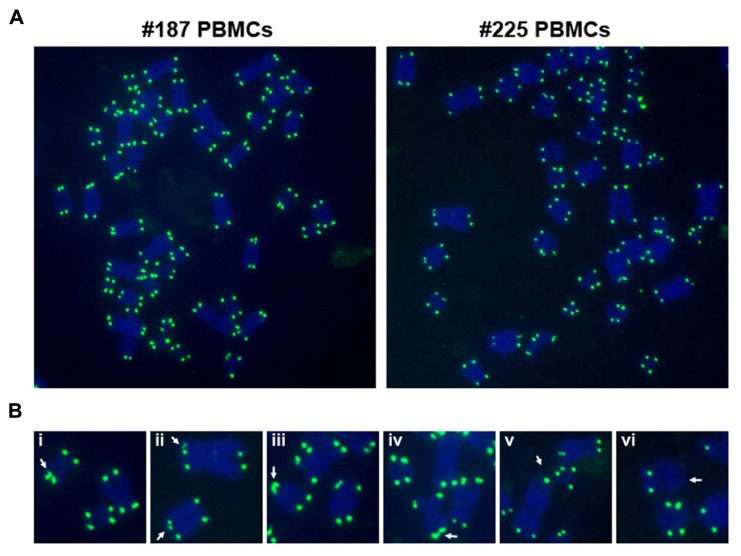
**EBV primary infection induces telomere dysfunction in peripheral blood mononuclear cells (PBMCs)**. **(A)** Freshly isolated PBMCs (#187 and #225) were infected with viruses isolated from Mutu I cells, and assayed by telomere DNA fluorescence *in situ* hybridization (FISH) on metaphase spreads at day 50 post-infection using telomeric PNA probe (green). Metaphase chromosomes were stained by Dapi, and shown in blue. **(B)** Common telomere aberrations in infected cells were shown as enlarged images. Arrows indicate telomere doublets (i–ii), telomere end fusions (iii–iv), and telomeric signal free ends (v–vi).

Viruses can also cause telomere dysfunction independently of telomerase activation. EBV infected cells have been shown to have dysfunctional telomeres (Kamranvar and Masucci, 2011; Kamranvar et al., 2007; Lacoste et al., 2009; **Figure 3**). EBNA1 has been implicated in the induction of telomere dysfunction through generation of reactive oxygen species (ROS) by transcription control of NOX2 (Gruhne et al., 2009; Kamranvar and Masucci, 2011). EBV-associated tumors, including EBV positive Reed-Sternberg cells in Hodgkin’s lymphoma may have altered telomere morphology and organization (Knecht et al., 2010a,b). Telomere clustering has been observed in several cancer cells, and virus induced proliferation may contribute to changes in telomere organization.

Viruses may also cause telomere dysfunction by integration into host telomeric DNA. HHV6 or MDV efficiently integrate into the host telomeric DNA through homologous recombination with the telomeric repeats at the viral termini. Viral TRs and telomere integration have a profound effect on MDV tumorigenesis and T-cell lymphomas. Although the potential direct effect on telomere dysfunction to MDV carcinogenesis is not known, telomere integration was shown to be important for efficient genome maintenance in infected cells (Kaufer et al., 2011b). Integration of HHV6 has no known pathology, but may correlate with cognitive and other neurological disorders. Whether this is due to telomere integration and telomere dysfunction is not yet known (Montoya et al., 2012).

Viral proteins can also bind telomeric factors and alter their ability to maintain telomere structure. HPV E6 has been shown to interact directly with the telomerase complex at telomeric DNA and this contributes to keratinocyte transformation by HPV (Liu et al., 2009). KSHV LANA has been shown to interact with both TRF1 and TRF2, and cause telomere shortening (Shamay et al., 2012). Consistent with LANA binding to TRF2 is the observation that TRF2 can also localize to the LANA-binding sites at the KSHV origin of replication (Hu et al., 2009). EBV EBNA1 was found to bind directly to Tankyrase, the TRF1-associated poly-ADP ribosylating enzyme (Deng et al., 2005). Tankyrase can modify EBNA1 and down-regulate its binding and function at OriP. Whether EBNA1 alters Tankyrase function at telomeres during EBV latent infection has not been determined. In summary, numerous interactions between viral proteins and host telomere regulatory factors have been reported. These reports underscore the significance of targeting telomeres and telomere maintenance mechanisms during viral infection.

## CONCLUSIONS

Viruses, like their hosts, actively and competitively maintain their genomes. In the process, virus infections may destabilizing host genomes with the consequence of cytopathic effects that can include carcinogenic insult. Many viruses, especially persistent DNA viruses, have specialized genome maintenance elements similar to host chromosomes. In this review, we have highlighted the numerous and diverse molecular mechanisms that contribute to TR stability, especially those that are shared by viruses and their host cells. Remarkably, these maintenance elements are themselves inherently unstable. Their repetitive nature makes them vulnerable to recombination and rearrangements. Their mechanisms of self-replication and post-replication processing are also threats to genetic stability. Understanding how these highly dynamic genetic elements balance genome stability with genome diversification is crucial to our understanding the forces that drive viral infection and cancer cell evolution. The knowledge gained from studying viral mechanisms of genome maintenance may provide insights into new anti-viral and anti-cancer therapies.

## Conflict of Interest Statement

The authors declare that the research was conducted in the absence of any commercial or financial relationships that could be construed as a potential conflict of interest.

## Abbreviations

AAV: adeno-associated virus
ATRX: alpha thalassemia/mental retardation syndrome X-linked
BNRF1: EBV major tegument protein
CTCF: CCCTC-binding factor
Daxx: death-domain associated protein
EBER: EBV-encoded RNA
EBNA1: EBV nuclear antigen 1
EBV: Epstein–Barr virus
ExoI: exonuclease I
HBV: hepatitis B virus
hCMV: human cytomegalovirus
HCV: hepatitis C virus
HHV6: human Herpesvirus 6
HPV: human papillomavirus
HSV: herpes simplex virus
hTERT: human telomerase reverse transcriptase
ICP0: infected cell polypeptide 0
IR: intergenic repeats
KSHV: Kaposi’s sarcoma-associated Herpesvirus
LANA: latency-associated nuclear antigen
LAT: HSV latency-associated transcript
LMP1 and 2: EBV latent membrane protein 1 and 2
MDV: Marek’s disease virus
ORC: origin recognition complex
Ori: viral replication origin
PML: promyelocytic leukemia
RCC1: regulator of chromosome condensation 1
TERRA: telomere repeats-containing RNA
TP: terminal-binding protein
TR: terminal repeats
TRF1 and 2: telomere repeat factor 1 and 2
XRCC4: X-ray repair cross-complementing protein 4.
